# Giant Cell Arteritis Presenting as Cholestatic Hepatitis

**DOI:** 10.1155/2021/4455748

**Published:** 2021-09-17

**Authors:** Gerald Riordan, John Riordan

**Affiliations:** ^1^Children's Hospital Westmead, Westmead, Australia; ^2^Illawarra Rheumatology, Wollongong, Australia

## Abstract

**Background:**

Severely deranged liver function tests (LFTs) are an atypical presentation of giant cell arteritis (GCA). Atypical presentations of GCA may result in missed or delayed diagnosis. This increases the risk of visual loss, the most feared outcome of GCA. Our patient presented with significant cholestatic derangement of his LFTs with a peak alkaline phosphatase level (ALP) of 3091 IU/L, which is the highest published level for patients with GCA. *Case Presentation*. Our patient was investigated for abnormal LFTs associated with sinus pain, fevers, and a dry cough. Bilateral temporal artery biopsies confirmed GCA. His symptoms and LFTs improved with corticosteroids.

**Conclusion:**

This is an unusual presentation of GCA and highlights the need to consider GCA in patients with unexplained cholestatic LFT abnormalities.

## 1. Introduction

A mild elevation in ALP occurs in 20–35% of patients with GCA [1]. There are two case reports of GCA causing significant LFT derangement; however, the peak ALP measured in these cases was 1017 IU/L [2]. To the best of our knowledge, the peak ALP of 3091 IU/L seen in this case is the highest recorded ALP caused by GCA. The pathophysiology of the raised ALP is unknown. Hepatic arteritis has been proposed by some authors, but remains unproven.

This case report is unique due to the severity of the LFT derangement. This is clinically important as it highlights the need for physicians to consider GCA when encountering abnormal LFTs. Particularly in the absence of typical GCA features, a wide differential for altered LFTs needs to be considered.

## 2. Case Presentation

An eighty-year-old man was admitted to hospital under hepatology for investigation of abnormal LFTs (ALP 2500 IU/L) associated with a raised C-reactive protein (CRP) (259 mg/L). He had a background of hypertension, hypercholesterolaemia, and gastro-oesophageal reflux. His regular medications were rabeprazole 20 mg/day, telmisartan 80 mg/day, amlodipine 10 mg/day, ezetimibe 10 mg/day, and aspirin 100 mg/day.

Six months prior, he had visited his primary physician with sinus pain. Blood tests taken at that time demonstrated an ALP of 215 IU/L (normal range 20–140 IU/L). He underwent removal of a left maxillary molar as a possible cause of his pain. However, he continued to experience sinus pain which was associated with head and neck pain, night sweats, and a dry cough. Blood tests then revealed an ALP of 2500 IU/L with a CRP of 259 mg/L and he was referred to hospital.

Examination on admission revealed mild nontender hepatomegaly with pitting oedema to his midshin. There were no cardiac murmurs. There was no tenderness over the maxillary sinuses and the dental socket was nontender. A transthoracic echo was unremarkable. A computer tomography coronary angiogram (CTCA) scan revealed borderline cardiac enlargement and a small pericardial effusion. A CT chest/abdomen/pelvis also demonstrated mild cardiac enlargement but had no other significant findings. An MRI cholangio-pancreatogram did not detect any biliary dilation or obstruction.

The patient's LFTs continued to deteriorate: ALP 3091 IU/L, GGT 753 IU/L, AST 147 IU/L, ALT 130 IU/L, and total bilirubin 16 *μ*mol/L. His ESR was also elevated (77 mm/hour) and his CRP remained elevated (250 mg/L). ANCA, ANA, anti-smooth muscle antibodies, liver/kidney antibodies, and viral serology were all negative.

In the context of raised inflammatory markers and atypical head and sinus pain, bilateral temporal artery biopsies were performed. Both biopsies demonstrated histological features of GCA, with giant cells and disruption of the internal elastic laminae. The patient was treated with oral corticosteroids and responded well to treatment. His LFTs normalised, his sinus pain abated, and his cough and oedema resolved.

## 3. Discussion and Conclusion

The typical symptoms of GCA include a subacute history of headaches, visual disturbances, jaw claudication, and fever. Uncommonly, GCA can cause peripheral oedema and upper respiratory tract symptoms, such as a nonproductive cough. It is also important to note that GCA can cause nonspecific prodromal symptoms (including weakness, fevers, and malaise). This highlights the need for clinicians to consider GCA even in the absence of classical features.

This case demonstrates that GCA can present atypically and the degree of LFT derangement can be significantly more severe than previously thought. Given the complications of delayed diagnosis of GCA (including blindness), it is important that physicians are aware of these clinical and biochemical features of GCA in order to facilitate diagnosis and treatment. Physicians need to maintain an open-minded consideration for GCA even in patients without typical GCA symptoms.

## Data Availability

Datasets are available on request from the corresponding author.

## Abbreviations

GCA: Giant cell arteritis
LFT: Liver function test
ALP: Alkaline phosphatase level
CRP: C-reactive protein
CTCA: Computer tomography coronary angiogram.
